# Supplementary material for: Illumina sequencing of 16S rRNA genes reveals a unique microbial community in three anaerobic sludge digesters of Dubai
Source: PLoS One. 2021 Apr 1;16(4):e0249023. doi: 10.1371/journal.pone.0249023 (PMC8016227; doi:10.1371/journal.pone.0249023)
Supplement: Attachment — Submitted filename: Authors response to Academic editor.docx [file pone.0249023.s004.docx]

Response to Additional Editor Comments

Journal Requirements:

When submitting your revision, we need you to address these additional requirements.

1. Please ensure that your manuscript meets PLOS ONE's style requirements, including those for file naming. The PLOS ONE style templates can be found at

<https://journals.plos.org/plosone/s/file?id=wjVg/PLOSOne_formatting_sample_main_body.pdf> and

<https://journals.plos.org/plosone/s/file?id=ba62/PLOSOne_formatting_sample_title_authors_affiliations.pdf>

**Response 1:** We have reformatted the manuscript to meet PLOS ONE's style requirements.

2. PLOS requires an ORCID iD for the corresponding author in Editorial Manager on papers submitted after December 6, 2016. Please ensure that you have an ORCID iD and that it is validated in Editorial Manager. To do this, go to ‘Update my Information’ (in the upper left-hand corner of the main menu), and click on the Fetch/Validate link next to the ORCID field. This will take you to the ORCID site and allow you to create a new iD or authenticate a pre-existing iD in Editorial Manager. Please see the following video for instructions on linking an ORCID iD to your Editorial Manager account: <https://www.youtube.com/watch?v=_xcclfuvtxQ>

**Response 2:** The corresponding author ORCID iD has been updated in the Editorial Manager.

3. Please include captions for your Supporting Information files at the end of your manuscript, and update any in-text citations to match accordingly. Please see our Supporting Information guidelines for more information: <http://journals.plos.org/plosone/s/supporting-information>.

**Response 3:** We have added supporting table information on the last page of the manuscript and amended in-text citations of supporting information according to the journal's guidelines.

4.Thank you for stating the following in the Funding Section of your manuscript:

"This work was supported by Zayed University Microbial Diversity and Ecogenomics Research Cluster Project (Grant code: R16092)."

We note that you have provided funding information that is not currently declared in your Funding Statement. However, funding information should not appear in the Acknowledgments section or other areas of your manuscript. We will only publish funding information present in the Funding Statement section of the online submission form.

Please remove any funding-related text from the manuscript and let us know how you would like to update your Funding Statement. Currently, your Funding Statement reads as follows:

 "The funders had no role in study design, data collection and analysis, decision to publish, or preparation of the manuscript."

Please include your amended statements within your cover letter; we will change the online submission form on your behalf.

**Response 4:** This is true that the funders had no role in study design, data collection and analysis, decision to publish, or preparation of the manuscript. However, the work would not have been possible without the generous financial support from the funding agency, that is why we want to acknowledge the funding agency. We have moved funding-related information to the acknowledgement section in the revised manuscript.

**Additional Editor Comments:**

**Point 1:** Both reviewers recommend rejection, but one of their key reasons for rejection, "significance", is not a valid reason for rejection from PLoS ONE. Moreover, I think that the reported data could be valuable contributions to the public databases and to the literature if properly discussed in the paper. Therefore, if you can make substantial revisions to the manuscript that address the reviewers' concerns, then I will consider a heavily revised manuscript.

**Response:** We appreciate the opportunity provided by the editor to considerably improve our manuscript for possible publication in PlosOne. Keeping in view the comments from the editor and reviewers the manuscript has been improved significantly. I hope the revised manuscript meets the standard of PlosOne and will be published in the journal.

**Point 2:** The revised manuscript must explain how the current study is related to previous work with the same or similar samples.

It must attempt to interpret the results from the perspective of trying to understand the microbial processes in anaerobic digester systems, rather than simply concluding that the microbial communities are "different". All microbial communities are different from each other. All microbial communities are unique. The revised manuscript should explain how the reported microbial diversity data helps to improve our knowledge of anaerobic digesters.

**Response:** The sludge samples for the next-generation sequencing analysis, FISH and qPCR analysis were collected simultaneously from the digesters. This information has been added to the revised manuscript. We agree to the editors comment, and we have elaborated the possible role of various microorganisms in the process of anaerobic digestion as also summarised in figure 4. Further information on the role of these microorganisms in the process of anaerobic digestion is also added to the revised manuscript, especially in the discussion section. We have concluded in the manuscript that the processes are mediated by the halophilic and/or thermophilic microorganisms which may be due to the influence of the local climatic condition. This study, therefore, is a primer for the possible role of microorganisms in the process of anaerobic digestion in gulf region. In future studies, we can try to isolate such halophilic or thermophilic bacteria from local environment that can help to improve the process of anaerobic digestion. In future we will do metagenome based study of the same digesters to understand the functional aspects more clearly.

**Point 3:** Finally, the authors should make their data publicly available. Currently, the provided BioProject accession is not associated with any sequence data that I can see.

The issues listed above are the minimum that should be addressed for a revised manuscript to be re-submitted. If the revision does not address all of the concerns from the reviewers, then it may be rejected even after revision. If you require additional time to make all necessary revisions, an extension can be requested.

**Response:** We have updated the deposited Nucleotide sequences files in the National Center for Biotechnology Information (Accession no. SRX10004771-SRX10004780) under the Bio-Project ID: PRJNA602372 on February 3, 2021. The sequence data records are now open to the public and accessible with the following link:

<https://www.ncbi.nlm.nih.gov/sra/PRJNA602372>
