# Supplementary material for: Illumina sequencing of 16S rRNA genes reveals a unique microbial community in three anaerobic sludge digesters of Dubai
Source: PLoS One. 2021 Apr 1;16(4):e0249023. doi: 10.1371/journal.pone.0249023 (PMC8016227; doi:10.1371/journal.pone.0249023)
Supplement: Attachment — Submitted filename: Author response to reviewers-2nd-revision.docx [file pone.0249023.s005.docx]

**Response to Reviewer’s /Additional Editor Comments**

**Comment**: Thank you for submitting your manuscript to PLOS ONE. After careful consideration, we feel that it has merit but does not fully meet PLOS ONE’s publication criteria as it currently stands. Therefore, we invite you to submit a revised version of the manuscript that addresses the points raised during the review process.

**Response:** Authors thank the reviewers for providing an opportunity to revise the manuscript. The manuscript is thoroughly revised keeping in view the suggestions of reviewers and academics editor. Manuscript is also considerably improved to avoid grammatical, language and editing mistakes.

**Comment**: Thank you for revising the manuscript. The revision addresses some but not all of the reviewer concerns. The responses to the reviewer's comments (e.g. the general importance of this study for effective wastewater management in Dubai and why these digesters were chosen) are not fully reflected in the text. Please ensure that the points made in your responses to the reviewers are also in the manuscript text.

**Response:** The manuscript is revised to include the importance of the study in the manuscript text. Furthermore, we have also modified the manuscript to include the changes suggested by reviewers earlier.

**Comment**: Also, Figure 4 contains a nice summary of some key results, but this figure is only briefly cited in the text. Please incorporate this figure more fully into the Results and Discussion.

**Response:** A detailed and duly discussion on figure 4 (now figure 2) is now included in the revised manuscript. Figure 4 is renamed as figure 2 to follow the in-text figure sequence style of the journal.

**Comment**: Finally, please check the entire manuscript for English grammar. For example, multiple sentences beginning with "Although" and "But" are not complete sentences.

**Response:** The manuscript is thoroughly checked for grammatical and editing mistakes by a native speaker. The authors hope that the language is significantly improved in the revised manuscript.
